# The Edible Plant *Crithmum maritimum* Shows Nutraceutical Properties by Targeting Energy Metabolism in Hepatic Cancer

**DOI:** 10.1007/s11130-022-00986-z

**Published:** 2022-07-14

**Authors:** Davide Gnocchi, Carlo Sabbà, Antonio Mazzocca

**Affiliations:** Interdisciplinary Department of Medicine, University of Bari School of Medicine, Piazza G. Cesare, 11, 70124 Bari, Italy

**Keywords:** *Crithmum maritimum* L., Edible wild plants, Hepatocellular carcinoma, Plant extracts, Bioenergetic phenotype, Anticancer activity

## Abstract

In the past few years, evidence has supported the role of plants as a valuable tool for the development of promising therapeutic support options for many diseases, including cancer. We recently discovered that the edible wild plant *Crithmum maritimum* L. effectively inhibits the growth of hepatocellular carcinoma (HCC) cells and we provide insights into the biological mechanisms involved. Here, we aimed to characterize the effect of ethyl acetate extract of *Crithmum maritimum* on the bioenergetic phenotype of HCC cells and if this is associated with the anti-tumour effect we previously described. Results show that *Crithmum maritimum* significantly increases cellular respiration and reduces lactic fermentation in HCC cells, and that this reduction of the fermentative glycolytic phenotype is linked to inhibition of HCC growth. These data provide new preclinical evidence supporting the role of *Crithmum maritimum* L. as a nutraceutical option to expand the therapeutic opportunities in the management of HCC.

**Supplementary Information:**

The online version contains supplementary material available at 10.1007/s11130-022-00986-z.

## Introduction

The utilisation of plant extracts and plant-derived products for therapeutic purposes is now gaining new importance for many pathological affections, including cancer [1]. Hepatocellular carcinoma (HCC) is nowadays the sixth leading cause of tumour-related deaths worldwide and expected as the third cause in Western countries by 2030 [2]. HCC management mainly relies on surgery, while the pharmacological approach to tyrosine-kinase inhibitors, such as sorafenib [3], is often combined with immunotherapy drugs. Such protocols have many adverse effects and cannot be tolerated in the long-range. Therefore, a novel effective, and better-tolerated therapeutic options for HCC are extremely needed.

*Crithmum maritimum* is known for a long time as an edible plant with general beneficial health effects, with antioxidant and antimicrobial properties [4]. Additional details on botanical, geographical and traditional medicinal and food uses are reported in Supplementary Material. We previously demonstrated that *Crithmum maritimum* ethyl acetate extract reduced HCC cell growth with low toxicity [5] and changed the metabolic profile, decreasing the level of choline and phosphocholine, lactate, amino acids, and cholesterol, directly involved in the control of cell growth [6] (Fig. S2b). This suggests that the cytostatic action exerted by *Crithmum maritimum* is at least in part due to the modulation of cellular metabolism. Also, *Crithmum maritimum* reduced the level of monounsaturated fatty acids (MUFA) and increased that of polyunsaturated fatty acids (PUFA) (Fig. S2b) [6]. We also provided preclinical evidence that *Crithmum maritimum* can be used in combination with sorafenib to reduce its dose and toxicity (Figure S3) [7].

We have been the first to describe and characterize the activity of *Crithmum maritimum* in inhibiting HCC growth [5], associating this effect with a complex multi-target action on HCC cell metabolism [6]. Here, we demonstrated that *Crithmum maritimum* changes the bioenergetic phenotype of HCC cells, activating oxidative phosphorylation (OXPHOS) and decreasing lactic fermentation. These observations support the view that activation of aerobic oxidative metabolism is associated with a reduction of tumour growth.

## Materials and Methods

The material and method section is reported in the Supplementary Material.

## Results and Discussion

**Fig. 1:**
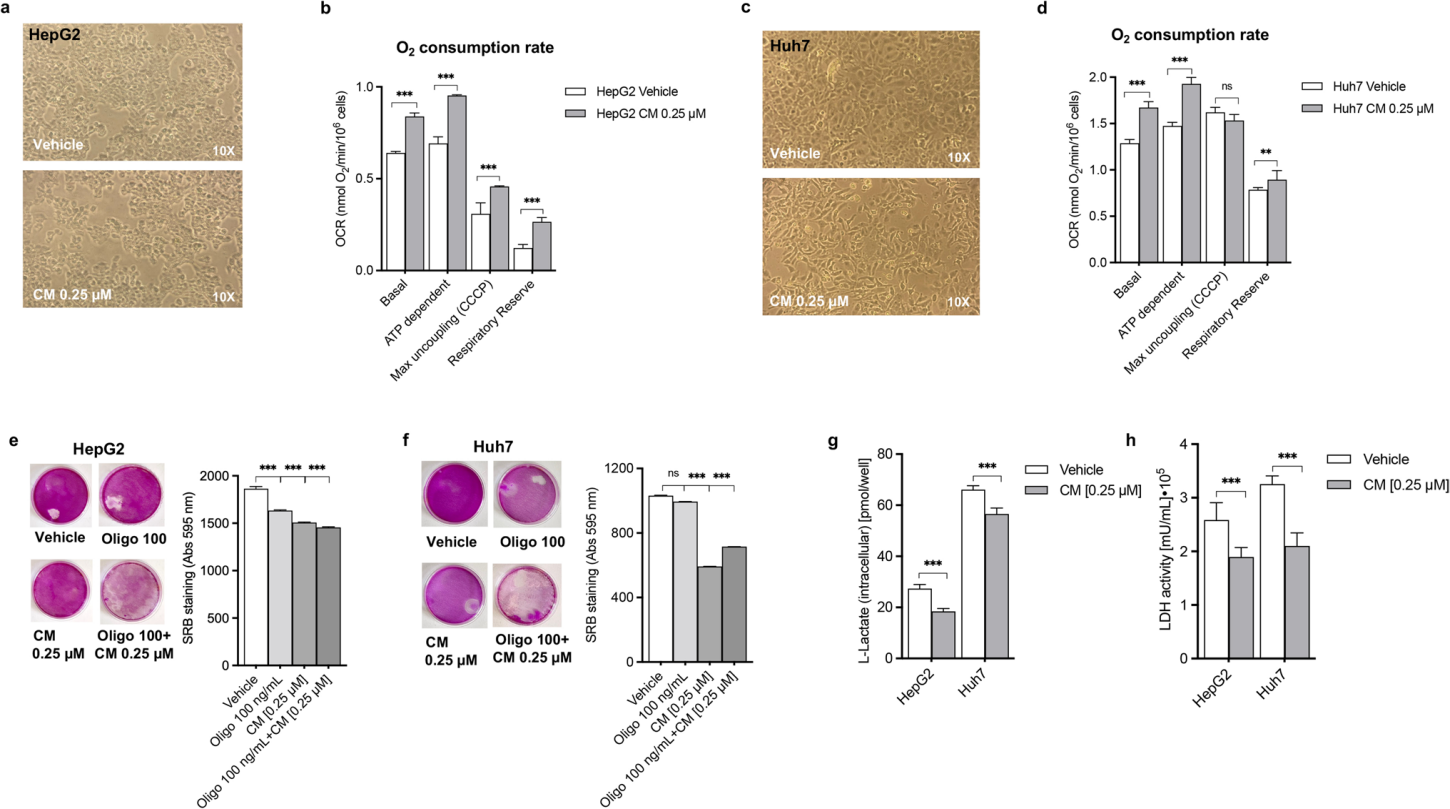
The inhibition of HCC cell growth by *Crithmum maritimum* (CM) ethyl acetate extracts in HCC cells (**a-c**) is associated with stimulation of OXPHOS (**b-d)**, increase of oligomycin sensitivity (**e-f)** and a reduction of lactic fermentation **(g-h)**. See text for more details. Data reported are representative of three independent experiments performed in duplicate. ns not significant, **p < 0.01, *** *p* < 0.001. Basal = Basal cellular respiration value; ATP dependent = Basal–Oligomycin values; Max uncoupling carbonyl cyanide m-chlorophenylhydrazone (CCCP) = CCCP value; Respiratory reserve = CCCP–Antimycin A value

We previously reported that *Crithmum maritimum* ethyl acetate extract effectively inhibits HCC cell growth [5] and determines a deep remodelling of the HCC cell metabolic profile [6]. Here, we aimed to characterise the role of *Crithmum maritimum* ethyl acetate extract on HCC cell bioenergetic phenotype and if this is associated with its anti-tumour effect [5, 6]. We assessed cellular respiration by employing a polarographic oxygraphy approach and determined intracellular lactate production and lactate dehydrogenase (LDH) activity. Figure 1a-c demonstrates the cytostatic effect after a 24 h treatment with *Crithmum maritimum* ethyl acetate extract in two HCC cell lines, HepG2 and Huh7. A 24 h treatment in the same conditions also determined a significant increase in basal respiration, ATP-dependent respiration and respiratory reserve, in both cell lines (Fig. 1b-d). Representative oxygraphy graphs are shown in Figure s4. More details about oxygraphic measurement can be found in Supplementary Material. The increased sensitivity of HCC cells to ATP synthase inhibitor oligomycin observed in oxygraphy measurements was confirmed by assessing oligomycin-induced cell toxicity after a 24 h pre-treatment with *Crithmum maritimum* (Fig. 1e-f). This corroborates the effects of *Crithmum maritimum* in activating ATP-dependent respiration. Figure 1b-d outlines the overall effect of *Crithmum maritimum* ethyl acetate extract on the activation of cellular respiration. Finally, Fig. 1g-h shows a significant decrease in intracellular lactate production and LDH activity after 24 h treatment with *Crithmum maritimum*. In summary, this supports the view that *Crithmum maritimum* shifts the bioenergetic phenotype of HCC cells from anaerobic glycolysis towards OXPHOS and this results in an inhibition of tumour growth.

## Conclusions

Based on our findings, we propose *Crithmum maritimum* as an effective choice for the development of new nutraceutical strategies in the treatment of HCC, aimed to support conventional HCC pharmacological therapies and to reduce side effects. Future research activity will be directed to the preparation of formulations suitable for human clinical trials to conduct case-control studies on HCC patients, combining appropriate extract preparations with standard therapies. Overall, we believe that this edible plant owns the potentiality to open the way for effective therapeutic options for HCC as well as for liver metabolic diseases, and possibly to other types of cancers.

## Supplementary Information

ESM 1 (PDF 4641 kb)

## Data Availability

Not applicable.

## References

[1] Newman DJ, Cragg GM (2020). Natural products as sources of new drugs over the nearly four decades from 01/1981 to 09/2019. J Nat Prod.

[2] de Lope CR, Tremosini S, Forner A, Reig M, Bruix J (2012). Management of HCC. J Hepatol.

[3] Forner A, Reig M, Bruix J (2018) Hepatocellular carcinoma. Lancet. 10.1016/S0140-6736(18)30010-2 10.1016/S0140-6736(18)30010-2 29307467

[4] Karkanis A, Polyzos N, Kompocholi M, Petropoulos SA (2022) Rock samphire, a candidate crop for saline agriculture: cropping practices, chemical composition and health effects. Appl Sci 12:737. 10.3390/app12020737

[5] Gnocchi D, Cesari G, Calabrese GJ, Capone R, Sabba C, Mazzocca A (2020). Inhibition of hepatocellular carcinoma growth by ethyl acetate extracts of Apulian Brassica oleracea L. and Crithmum maritimum L. Plant Foods Hum Nutr.

[6] Gnocchi D, Del Coco L, Girelli CR, Castellaneta F, Cesari G, Sabba C, Fanizzi FP, Mazzocca A (2021). (1)H-NMR metabolomics reveals a multitarget action of Crithmum maritimum ethyl acetate extract in inhibiting hepatocellular carcinoma cell growth. Sci Rep.

[7] Gnocchi D, Castellaneta F, Cesari G, Fiore G, Sabba C, Mazzocca A (2021) Treatment of liver cancer cells with ethyl acetate extract of *Crithmum maritimum* permits reducing sorafenib dose and toxicity maintaining its efficacy. J Pharm Pharmacol. 10.1093/jpp/rgab070 10.1093/jpp/rgab070 34014301

