# Supplementary material for: The Edible Plant Crithmum maritimum Shows Nutraceutical Properties by Targeting Energy Metabolism in Hepatic Cancer
Source: Plant Foods Hum Nutr. 2022 Jul 14;77(3):481–3. doi: 10.1007/s11130-022-00986-z (PMC9463332; doi:10.1007/s11130-022-00986-z)
Supplement: Supplementary file 1 — (PDF 4641 kb) [file 11130_2022_986_MOESM1_ESM.pdf]

__Short Communication – Supplementary Material__

The edible plant *Crithmum maritimum* shows nutraceutical properties by targeting energy metabolism in hepatic cancer

Davide Gnocchi[a], Carlo Sabbà[a], Antonio Mazzocca[a*]

[a] Interdisciplinary Department of Medicine, University of Bari School of Medicine, Piazza G. Cesare, 11 - 70124 Bari, Italy

[*] Corresponding author:

Antonio Mazzocca, M.D., Ph.D., Interdisciplinary Department of Medicine, University of Bari School of Medicine, Piazza G. Cesare, 11 I-70124 Bari, Italy

Tel.: +39 080 5593593

E-mail address: antonio.mazzocca@uniba.it

Supplementary Text

Botanical classification and geographical distribution

Crithmum maritimum is a perennial herbaceous plant, which belongs to the family of *Umbelliferae* or *Apiaceae*, the only species of the genus *Crithmum*. The present name derives from the Greek word “Cretzmon”, whose origin was attributed or to the leaves’ shape or to the fact that the seed resembles that of barley [1,2]. It has many popular names, the most common of them being “rock samphire, samphire, marine fennel, crest marine” in English. As an indication of the wide and old tradition of this plant, there are many popular names also in other languages. In France, *Crithmum maritimum* is known as “perce-pierre, passepierre, fenouil marine, criste-marine”. In Germany as “Seefenchel, Meerfenchel”, and in Spain as “perejil marino, hinojo marino”. In Italy, *Crithmum maritimum* has different designations in different regions of the country: “finocchio marino, erba di S. Pietro, cretamo, critama, bacicci, basiggia and spaccasassi” [3]. From a botanical point of view, *Crithmum maritimum* is a perennial halophyte that finds its habitat along coastlines, where it grows on or among rocks, seawalls as well as on beaches (Fig. S1).

a **b**

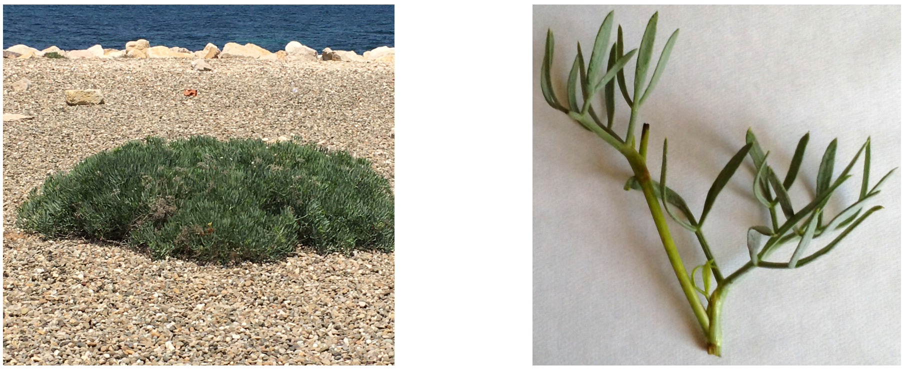

Fig. S1 Pictures of *Crithmum maritimum* L. collected on the coastlines around Bari, Apulia (Italy). (**a**) Bushes of *Crithmum maritimum* L. in proximity of the sea (**b**) Branch of *Crithmum maritimum* L.

Regarding geographical distribution, *Crithmum maritimum* is endemic in North Africa, Mediterranean countries, to France, British Islands and Ireland. It was also described on the Pacific coast [4,2]. *Crithmum maritimum* plants have a branched bushy appearance and can reach a maximum height of about 60 cm (Fig. S1). Leaves are small and succulent, flowers develop at the beginning of summer (July) and last until the end of summer (September), while fruits are usually produced in October-November and ripe until December [1,2].

Traditional and recent usage of *Crithmum maritimum*

Historical perspective

Crithmum maritimum is known from ancient times. Indeed, it is mentioned in Hippocrates' "Corpus Hippocraticum" as well as in Plinius' "Historia Naturalis", where it is suggested as a good remedy for renal conditions and gout [5]: "The Critmo, is highly praised by Hippocrates. It is one of those wild herbs that are eaten and it is certainly this, that according to Callímaco, is served by the farmer Ecale to Theseus". Later, in the Byzantine period, it is described with the name "crithmom" in the "Dynameron", a medical manuscript written by Nikolaos Myrepsos [6]. Interestingly, *Crithmum maritimum* is also mentioned in Shakespeare's "King Lear" (Act 4, scene 6), where it is written, …" it is terrible job… The habit of climbing the cliffs to collect it because often halfway, those who search for it, fall"…

Traditional medicinal and food uses

Populations living along the Mediterranean Sea recognised and attributed to *Crithmum maritimum* many medicinal properties, such as diuretic and depurative, digestive, antiscorbutic, anti-cold and anti-inflammatory, and also wound healing and vermifuge properties (Table 1) [2].

Table 1
Crithmum maritimum traditional uses

Properties	Regional uses	References
Diuretic, depurative, digestive	Mediterranean countries, Italy, Spain	Atia et al., 2011a; Carrio and Valles, 2012
Antiscorbutic	Mediterranean countries	Atia et al., 2011a
Anti-cold, anti-cough	Italy	Savo et al., 2011
Anti-bacterial, anti-mycotic, vermifuge	Mediterranean countries	Senatore et al., 2000; Glamoclija et al., 2009
Prostatitis, cystitis	Italy	Atia et al., 2011a
Liver functionality	Italy	Cornara et al., 2009
Insecticidal	Mediterranean countries	Pavela et al., 2017; Polatoglu et al., 2016; Tsoukatou et al., 2001

Also, fishermen were used to eat *Crithmum maritimum* leaves as a remedy to scurvy, and the Italian tradition describes *Crithmum maritimum* infusion as an adjuvant for digestion, while the decoction was employed for prostatitis and cystitis [2]. In Spain, pickled leaves are still considered a good way to improve digestion and as a diuretic [7]. In the Easter Riviera in Liguria (Italy), *Crithmum maritimum* decoction is used as an adjuvant of liver functionality [8], whereas in the South of Italy (Amalfi coast) is employed to relieve the symptoms of cold and cough [9]. Of note, *Crithmum maritimum* essential oil was shown to own antibacterial [10] and antimycotic [11] activity. Interestingly, the effect of *Crithmum maritimum* as an insecticidal agent was reported against different genera of insects [12-14].
Crithmum maritimum is very appreciated also as a food. The leaves are eaten as a salad or cooked or also treated in vinegar similarly to capers. This preparation in Apulia is included in the Ministry of Agriculture list of traditional agri-food products [4]. In the United Kingdom, leaves and stems of *Crithmum maritimum* are cooked together with pickled cucumber and capers to obtain the so-called "Rock Samphire hash" [4].

Phytochemical characteristics

The phytochemical characterisation of the plant, which has been performed during the last decades, has provided some scientific basis to these folk medicine traditions and usages (Table 2).

Table 2
Crithmum maritimum phytochemical characteristics

Compounds/Activity	Plant prevalent localization site/Physiological Stage	References
Vitamin C, flavonoids, carotenoids	Leaves/vegetative stage	Ben Hamed et al., 2005
Antioxidant activity	Leaves/vegetative stage	Jallali et al., 2012
phenolic compounds, flavonoids	Leaves/vegetative & flowering stage	Jallali et al., 2012
Phenolic compounds (chlorogenic acid, quinic acid and caffeoylquinic acid)	Leaves/vegetative stage	Meot-Duros and Magne, 2009; Nabet et al., 2017
Essential fatty acids	seeds	Ben Hamed et al., 2005
Volatile compound (terpenoids)	shoots and fruits	Atia et al., 2011a

The leaves of *Crithmum maritimum* have a high content of Vitamin C, flavonoids and carotenoids, as well as of several classes of bioactive compounds. Moreover, from its seeds, it is possible to extract an edible oil, which was recognized as a good source of essential fatty acids [15]. For what concerns bioactive compounds, reported quantifications to differ among different studies. This is most probably due to the different protocols employed, as well as to the growing area and the period of the year [16]. It was described that the phenolic composition and antioxidant activity of *Crithmum maritimum* are different between vegetative and flowering physiological stages. Interestingly, the antioxidant activity was significantly higher in the vegetative stage compared to the flowering stage, while the amount of phenolic compounds and flavonoids did not significantly differ between the two physiological stages [17]. About the phenolic compound composition, some studies reported that *Crithmum maritimum* is rich in chlorogenic acid, quinic acid and caffeoylquinic acid. A higher concentration of these compounds was found in leaves compared to flowers and stems [18,19]. The presence of several volatile compounds, mostly terpenoids, was also described, prevalently in shoots and fruits [2].

The metabolite profile of the dried powder of *Crithmum maritimum* we employed in our studies was determined by employing ^{1}H-NMR metabolomics. The powder was found to be particularly rich in chlorogenic acids, while the ethyl acetate extract was mainly composed of falcarindiol, flavonoids, carotenoids and hydroxycinnamates, and hydroxybenzoic acid derivatives (Table 3).

Table 3
Crithmum maritimum ethyl acetate extract composition

Classes of compounds	Compounds	Structure of representative compounds
Fatty acids, sterols, di- and tri-acylglycerols	β-sitosterol; stigmasterol; ω3-fatty acids (linolenic acid); ω6-fatty acids (linoleic acid); glycerolipids (digalactosyldiacylglycerol)	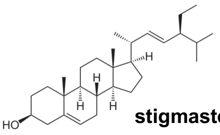 **stigmasterol** 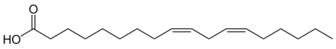 **linolenic acid**
Polyacetylenes	falcarindiol	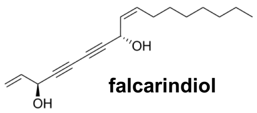 **falcarindiol**
Phenolic compounds	carotenoids; hydroxycinnamic acids (chlorogenic acid); hydroxybenzoic acid derivatives (epigallocatechin H2 and H6, rutin, apigenin, catechin, epicatechin, and quercetin)	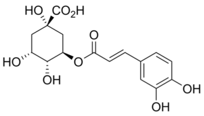 **chlorogenic acid** 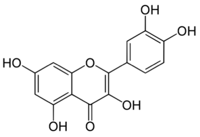 **quercetin**
Photosynthetic pigments	chlorophylls (chlorophyll a and b) pheophytins	**chlorophyll a** 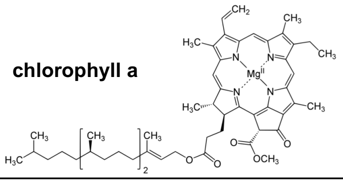

Materials and Methods

Cell lines

HepG2 and Huh7 cell lines were obtained from the JCRB cell bank [cat. # JCRB0403 and cat. # JCRB1054, respectively]. Both cell lines were grown in Dulbecco's modified Eagle's medium (DMEM) with 1 g/L glucose, 4 mM glutamine, and 1 mM sodium pyruvate [Corning cat. #10-014-CVR], supplemented with 10% Foetal Bovine Serum (FBS) [Corning cat. # 35-079-CV], 1X MEM-Nonessential Amino Acids [Corning cat. # 25-025-CIR], 20 mM Hepes Buffer [Aurogene cat. # AU-L0180-500], 1X Antibiotic-Antimycotic solution [Corning cat. # 30-004-CI]. Cells were cultured under standard conditions (humidified atmosphere, 37°C and 5% CO_2).

Microscope pictures

Pictures were taken with a Nikon TMS (model DS-Fi1) associated with a "Nikon Digital Sight" digital camera and with "NIS-Elements F3.0" software. Pictures reported were taken at 10X magnification.

Measurement of oxygen consumption

Oxygen consumption was measured with a polarographic approach using a Clark-type oxygen electrode in a water-jacketed chamber (Hansatech Instruments, Norfolk, UK) as previously described [20]. Briefly, after trypsinization, cells were resuspended in a specific "respiration" buffer (buffer A= 75 mM sucrose, 5 mM KH_2PO_4, 40 mM KCl, 0.5 mM EDTA, 3 mM $MgCl_2$, 30 mM Tris-HCl, pH 7.4) and inserted inside the water-jacketed chamber to get the final volume of 1 mL. Results are reported as nmol O_2/mL/min/10^6 cells. For the determination of the "respiratory fingerprint", cells were inserted in the oxygraphic chamber and treated with oligomycin (2 μg/μL), CCCP (1 μM), antimycin A (15 nM), and rotenone (1 μM). Basal = Basal cellular respiration value; ATP dependent = Basal–Oligomycin values; Max uncoupling (CCCP) = CCCP value; Respiratory Reserve = CCCP–Antimycin A value. Oligomycin: Inhibitor of complex V of mitochondrial oxidative phosphorylation (ATP synthase). CCCP: carbonyl cyanide m-chlorophenylhydrazone, an uncoupler of mitochondrial oxidative phosphorylation; Antimycin A: Inhibitor of complex III of mitochondrial oxidative phosphorylation.

Sulphorhodamine B (SRB) assay

Preliminarily cells were fixed by adding a Fixative Reagent (50% Trichloroacetic acid v/v in ultrapure distilled water) directly in the culture media in a 1:4 ratio. After a 1 h fixation at 4°C, cells were washed four times with ultrapure distilled water. Once cells were dried, the Sulphorhodamine B solution (Sulphorhodamine B solution salt, Sigma-Aldrich cat. #S1402, 0.4% in 1% v/v acetic acid in ultrapure distilled water) was added and allowed to stain for at least 30 minutes in the dark. After removing the staining solution, cells were washed four times with a Wash Solution (1% v/v

acetic acid in ultrapure distilled water). Once cells were dried, the bound SRB was solubilized using an SRB Solubilization Buffer (Tris base 10 mM pH 10.5). Absorbance was read with an iMark™ plate reader at λ=595 nm.

Determination of intracellular lactate production

Intracellular lactate production was assessed by employing an enzymatic colorimetric kit [Sigma-Aldrich cat. # MAK064]. Cells were homogenized in an assay buffer and after centrifugation, endogenous lactate dehydrogenase was removed by centrifugation using Amicon Ultra-0.5 Centrifugal Filter Units [Merck cat. # UFC501096]. After incubation with substrate and enzyme provided by the kit, absorbance was measured at 490 nm. Lactate concentration was determined by interpolation with a standard curve.

Determination of lactate dehydrogenase (LDH) activity

Lactate dehydrogenase activity was determined by using a colorimetric enzymatic kit [Sigma-Aldrich cat. #MAK066]. Briefly, cells were homogenized in an Assay Buffer and, after centrifugation, the supernatant was used for the assay. Absorbance, measured at 450 nm, is proportional to the concentration of NADH produced, which was determined by interpolation with a standard curve. LDH activity was then calculated by employing the following equation: LDH activity = F/(Reaction Time)*V where F=amount of NADH generated in the interval of time considered and V=sample volume (mL) in the well. Results are reported as mU/mL where 1 U of LDH = the amount of the enzyme that catalyses the conversion of lactate-pyruvate to generate 1.0 μmol of NADH/min at 37°C.

Statistical analyses

The normality of data was verified with D'Agostino-Pearson's Omnibus K2 test. One-Way ANOVA followed by Dunnett's post-hoc test was used to determine statistical significance when data were normally distributed.

Supplementary Figures

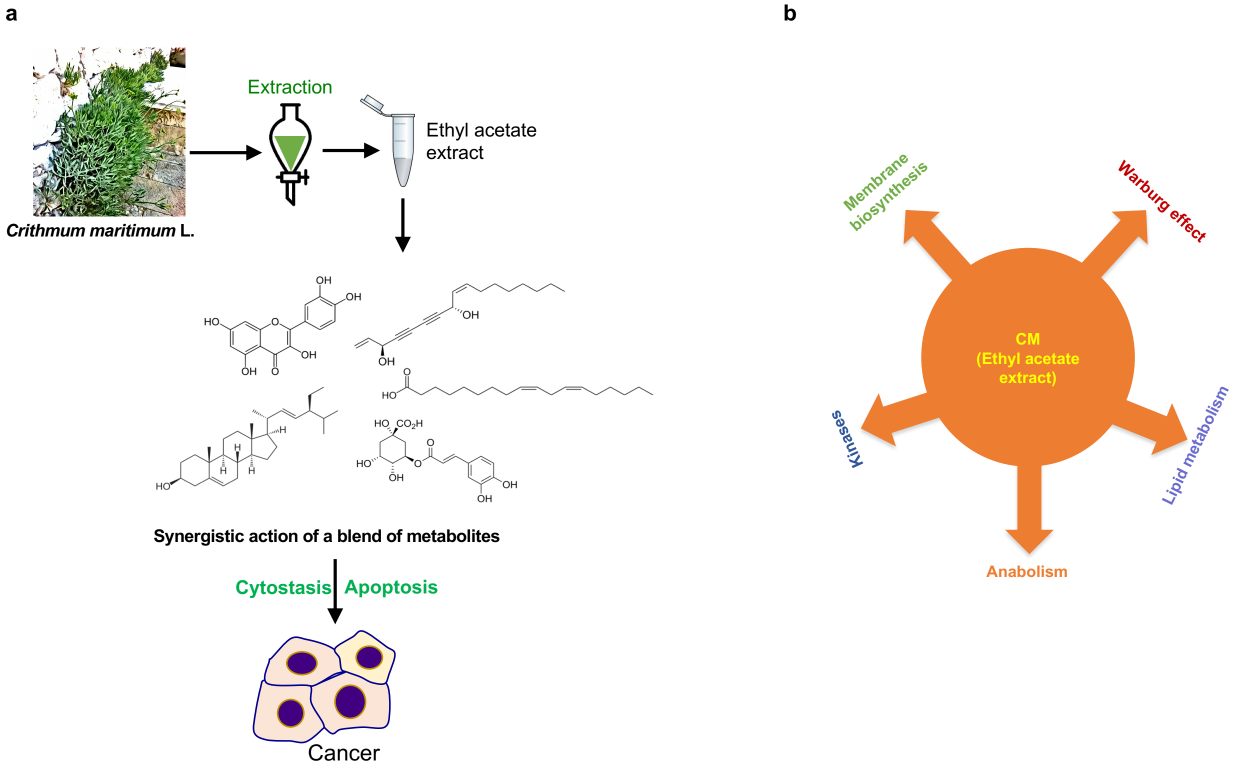

Fig. S2 Schematic diagram summarizing the main findings of our previous research on *Crithmum maritimum* L. (**a**) Experimental workflow from obtainment of the ethyl acetate active fraction to the observed cytostatic effect on HCC cells. (**b**) Schematic diagram outlining the multi-target effect induced by *Crithmum maritimum* L. in HCC cells that explains the cytostatic effects observed.

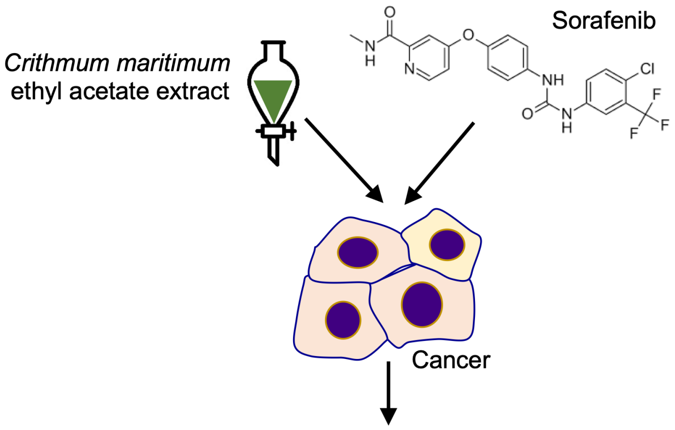

Treatment	Efficacy	Toxicity
Sorafenib (full-dose)	* * *	* * *
CM ethyl acetate extract	* *	*
Sorafenib (half-dose)+CM ethyl acetate extract	* * *	*

Fig. S3 Schematic diagram representing our previous research on the effect of the combined treatment of HCC cells with half dose sorafenib and *Crithmum maritimum* L. ethyl acetate extract, showing that the combination can reduce toxicity while maintaining efficacy.

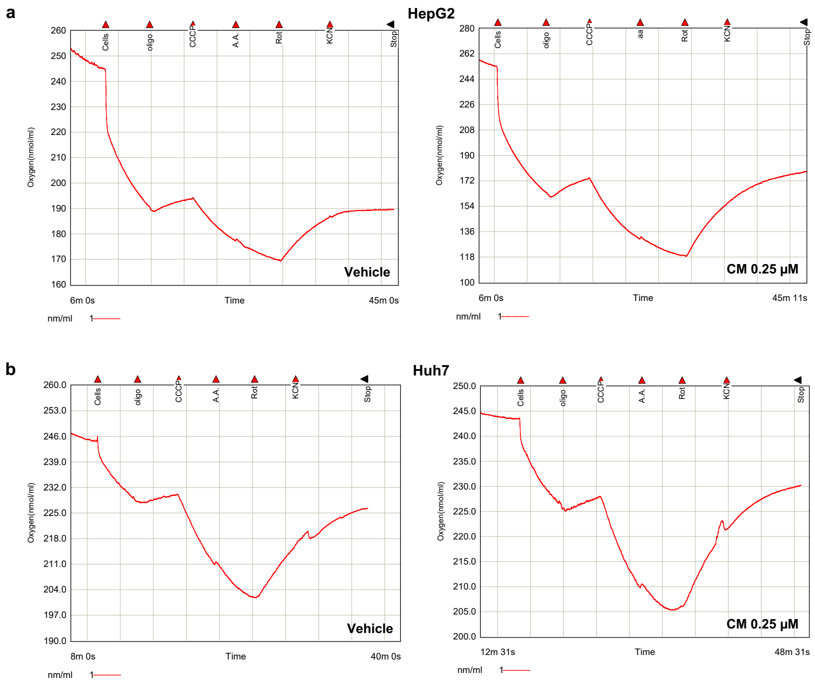

Fig. S4 Representative oxygraphic graphs in HepG2 (a) and Huh7 (b) cells.

Supplementary References

1. Atia A, Chokri H, Mokded R, Barhoumi Z, Abdelly C, Smaoui A (2011) Anatomy of the fruit of the halophyte Crithmum maritimum L. with emphasis on the endosperm structure and histochemistry. Afr J Biotechnol 10 (45):9193-9199

2. Atia A, Barhoumi Z, Mokded R, Abdelly C, Smaoui A (2011) Environmental eco-physiology and economical potential of the halophyte Crithmum maritimum L. (Apiaceae). J Med Plants Res 5 (16):3564-3571

3. Renna M (2018) Reviewing the Prospects of Sea Fennel (Crithmum maritimum L.) as Emerging Vegetable Crop. Plants (Basel) 7 (4). doi:10.3390/plants7040092

4. Renna M, Gonnella M, Caretto S, Mita G, Serio F (2017) Sea fennel (Crithmum maritimum L.): from underutilized crop to new dried product for food use. Genet Resour Crop Ev 64 (1):205-216. doi:10.1007/s10722-016-0472-2

5. Aliotta G, Pollio A (1994) Useful plants in renal therapy according to Pliny the Elder. Am J Nephrol 14 (4-6):399-341. doi:10.1159/000168755

6. Coiffard L, Piron-Frenet M, Amicel L (1993) Geographical variations of the constituents of the essential oil of Crithmum maritimum L., Apiaceae. Int J Cosmet Sci 15 (1):15-21. doi:10.1111/j.1467-2494.1993.tb00064.x

7. Carrio E, Valles J (2012) Ethnobotany of medicinal plants used in Eastern Mallorca (Balearic Islands, Mediterranean Sea). J Ethnopharmacol 141 (3):1021-1040. doi:10.1016/j.jep.2012.03.049

8. Cornara L, La Rocca A, Marsili S, Mariotti MG (2009) Traditional uses of plants in the Eastern Riviera (Liguria, Italy). J Ethnopharmacol 125 (1):16-30. doi:10.1016/j.jep.2009.06.021

9. Savo V, Giulia C, Maria GP, David R (2011) Folk phytotherapy of the Amalfi Coast (Campania, Southern Italy). J Ethnopharmacol 135 (2):376-392. doi:10.1016/j.jep.2011.03.027

10. Senatore F, Napolitano F, Ozcan M (2000) Composition and antibacterial activity of the essential oil from Crithmum maritimum L. (Apiaceae) growing wild in Turkey. Flavour Frag J 15 (3):186-189. doi:Doi 10.1002/1099-1026(200005/06)15:3<186::Aid-Ffj889>3.0.Co;2-I
11. Glamoclija J, Sokovic M, Grubisic D, Vukojevic J, Milinekovic I, Ristic M (2009) Antifungal activity of Critmum maritimum essential oil and its components against mushroom pathogen Mycogone perniciosa. Chem Nat Compd+ 45 (1):96-97. doi:10.1007/s10600-009-9242-0
12. Tsoukatou M, Tsitsimpikou C, Vagias C, Roussis V (2001) Chemical intra-Mediterranean variation and insecticidal activity of Crithmum maritimum. Z Naturforsch C 56 (3-4):211-215
13. Polatoglu K, Karakoc OC, Yucel YY, Gucel S, Demirci B, Baser KHC, Demirci F (2016) Insecticidal activity of edible Crithmum maritimum L. essential oil against Coleopteran and Lepidopteran insects. Ind Crop Prod 89:383-389. doi:10.1016/j.indcrop.2016.05.032
14. Pavela R, Maggi F, Lupidi G, Cianfaglione K, Dauvergne X, Bruno M, Benelli G (2017) Efficacy of sea fennel (Crithmum maritimum L., Apiaceae) essential oils against Culex quinquefasciatus Say and Spodoptera littoralis (Boisd.). Ind Crop Prod 109:603-610. doi:10.1016/j.indcrop.2017.09.013
15. Ben Hamed K, Ben Youssef N, Ranieri A, Zarrouk M, Abdelly C (2005) Changes in content and fatty acid profiles of total lipids and sulfolipids in the halophyte Crithmum maritimum under salt stress. Journal of Plant Physiology 162 (5):599-602. doi:10.1016/j.jplph.2004.11.010
16. Mekinic IG, Simat V, Ljubenkov I, Burcul F, Grga M, Mihajlovski M, Loncar R, Katalinic V, Skroza D (2018) Influence of the vegetation period on sea fennel, Crithmum maritimum L. (Apiaceae), phenolic composition, antioxidant and anticholinesterase activities. Ind Crop Prod 124:947-953. doi:10.1016/j.indcrop.2018.08.080
17. Jallali I, Megdiche W, M'Hamdi B, Oueslati S, Smaoui A, Abdelly C, Ksouri R (2012) Changes in phenolic composition and antioxidant activities of the edible halophyte Crithmum maritimum L. with physiological stage and extraction method. Acta Physiol Plant 34 (4):1451-1459. doi:10.1007/s11738-012-0943-9

18. Nabet N, Boudries H, Chougui N, Loupassaki S, Souagui S, Burlo F, Hernandez F, Carbonell-Barrachina AA, Madani K, Larbat R (2017) Biological activities and secondary compound composition from Crithmum maritimum aerial parts. Int J Food Prop 20 (8):1843-1855. doi:10.1080/10942912.2016.1222541

19. Meot-Duros L, Magne C (2009) Antioxidant activity and phenol content of Crithmum maritimum L. leaves. Plant Physiol Biochem 47 (1):37-41. doi:10.1016/j.plaphy.2008.09.006

20. Pacelli C, Latorre D, Cocco T, Capuano F, Kukat C, Seibel P, Villani G (2011) Tight control of mitochondrial membrane potential by cytochrome c oxidase. Mitochondrion 11 (2):334-341. doi:10.1016/j.mito.2010.12.004
